# Does Intracellular Metabolism Render Gemcitabine Uptake Undetectable in Mass Spectrometry?

**DOI:** 10.3390/ijms23094690

**Published:** 2022-04-23

**Authors:** Julian Peter Müller, Dirk Gründemann

**Affiliations:** Department of Pharmacology, Faculty of Medicine and University Hospital Cologne, University of Cologne, Gleueler Straße 24, 50931 Cologne, Germany; mueller.julian-p@gmx.de

**Keywords:** *SLC22A4*, ergothioneine, ergothioneine transporter, drug transport, nucleoside transport, nucleoside metabolism, CNT3

## Abstract

The ergothioneine transporter ETT (formerly OCTN1; human gene symbol *SLC22A4*) is a powerful and highly specific transporter for the uptake of ergothioneine (ET). Recently, Sparreboom et al. reported that the ETT would transport nucleosides and nucleoside analogues such as cytarabine and gemcitabine with the highest efficiency. In our assay system, we could not detect any such transport. Subsequently, Sparreboom suggested that the intracellular metabolization of the nucleosides occurs so fast that the original compounds cannot be detected by LC–MS/MS after inward transport. Our current experiments with 293 cells disprove this hypothesis. Uptake of gemcitabine was easily detected by LC–MS/MS measurements when we expressed the Na^+^/nucleoside cotransporter CNT3 (*SLC28A3*). Inward transport was 1280 times faster than the intracellular production of gemcitabine triphosphate. The deoxycytidine kinase inhibitor 2-thio-2′-deoxycytidine markedly blocked the production of gemcitabine triphosphate. There was no concomitant surge in intracellular gemcitabine, however. This does not fit the rapid phosphorylation of gemcitabine. Uptake of cytarabine was very slow, but detection by MS was still possible. When the ETT was expressed and incubated with gemcitabine, there was no increase in intracellular gemcitabine triphosphate. We conclude that the ETT does not transport nucleosides.

## 1. Introduction

Nucleoside transporters are interesting drug targets [1,2,3,4,5,6]. For example, inhibition of the equilibrative nucleoside transporter 1 (ENT1, *SLC29A1*) is a potential treatment for ischemic heart disease, stroke, and cancer [7]. Dilazep and dipyridamole, the two marketed non-selective ENT1 inhibitors, are supposed to cause vasodilation by increasing the extracellular concentration of adenosine. The equilibrative nucleoside transporters (ENTs) belong to the *SLC29*, and the concentrative nucleoside transporters (CNTs), to the *SLC28* gene family, respectively. Several of those catalyze the transport of important anti-cancer nucleoside drugs such as cytarabine and gemcitabine.

A recent report published in *Cancer Research* claimed that the ergothioneine transporter (ETT; obsolete name OCTN1) would transport nucleosides and nucleoside analogues with the highest efficiency and, thus, represent an important novel nucleoside transporter [8]. Very high clearance values were reported for cytarabine (ca. 180 µL min^−1^ mg protein^−1^) and gemcitabine (ca. 130 µL min^−1^ mg protein^−1^) (external Figure 5B; in this paper, “external” indicates material from the cited study), absolutely comparable to the transport efficiency (TE) for ergothioneine (50 to 200 µL min^−1^ mg protein^−1^).

Ergothioneine (ET) is a very hydrophilic zwitterion at physiological pH; it cannot penetrate cell membranes without help. The discovery of the evolutionarily conserved (ETT) gave a powerful boost to the expectation of a beneficial role for ET [9]. The Na^+^-driven ETT is highly selective and highly efficient for the uptake of ET into cells [10]. The ETT was the first and so far only biomarker for ET activity [11]: Only cells with strong expression of ETT at the surface can accumulate ET to high levels. The product of the human gene *SLC22A4* is responsible for the uptake of ET from the diet, for its distribution in the body, and for its recovery from the primary urine in the kidney [10].

In our heterologous expression system, we could not detect any transport by human and rat ETT with cytarabine, gemcitabine, 2′-deoxycytidine, and 2′-deoxyadenosine [12]. The accumulation of a compound in cells expressing human or rat ETT was not higher than that in control cells. In such experiments, we use regulated expression (inducible by the addition of doxycycline to the culture medium) of the transporter in 293 cells with a plasmid that is not integrated into the genome of the cells [13]. Normally, we quantify unlabeled substrates by LC–MS/MS, with the advantage of high specificity, since in the selected reaction mode (SRM), molecules are detected only when they pass two separate mass filters.

The reason for the striking discrepancy between our results and those of Sparreboom et al. remained unclear. Peculiarly, the inhibition profile of nucleoside transport by the ETT (with dipyridamole 10 times more potent than NBMPR, but the IC_50_ of NBMPR is still below 0.1 µM) reported by Sparreboom et al. [8] did not match any of the known nucleoside transporters. The uptake of cytarabine (1 µM) was not inhibited in the slightest by ET (the concentration was roughly reported as 10–50 µM; external Figure 3A), which would imply a second binding site for nucleosides, separate from the binding site for ET [8]. The concept of a transporter with two distinct binding sites for different substrates is not completely unreasonable, as exemplified by OAT2 (*SLC22A7*), which transports both glutamate and orotic acid [14], and by SLC22A13 [15].

Meanwhile, Sparreboom et al. continued to consider the ETT as a nucleoside transporter [16,17]. In their own LC–MS/MS measurements, they also failed to detect an increase in intracellular cytarabine; however, using a radiotracer, a high uptake was again registered in parallel [16]. Their explanation was that, after transport by the ETT into cells, the nucleoside is phosphorylated so rapidly that the original compound cannot be detected in MS [16].

The very rapid intracellular metabolization of nucleosides has indeed been reported elsewhere [18,19], so the argument cannot be dismissed out of hand. The aim of the current research was, therefore, to test the hypothesis that metabolization is much faster than transport, so that the compound because of the mass change inherent in phosphorylation escapes mass spectrometry set to detect the unmodified compound. To this end, we expressed CNT3 as a model gemcitabine transporter in 293 cells and could indeed measure the uptake of non-metabolized gemcitabine and cytarabine. Moreover, the intracellular generation of gemcitabine triphosphate was stimulated by CNT3-catalyzed gemcitabine uptake but not by the expression of the ETT.

The 293 cells, popular for heterologous expression of transporters, are actually less suitable for measuring the uptake of nucleosides because of the presence of endogenous transporters. Cell lines such as PK15/NTD without endogenous uptake would serve much better [20]. Although PK15/NTD was available to Sparreboom et al. (external Figure 3B), most measurements of uptake of cytarabine and gemcitabine were performed with 293 cells [8]. We, therefore, also used 293 cells for a comparison of transport and metabolism. Gemcitabine was our chief substrate. Relevant plasma membrane transporters for gemcitabine are ENT1, ENT2, CNT1, and CNT3 [21]. From this group, ENT1 is numerically most abundant in 293 cells, with approximately 5× [21] and 3.8× (external Figure S1c in [8]) more mRNA than ENT2, respectively. CNT1 and CNT3 are virtually not expressed in 293 cells, with 1000× less mRNA than ENT1 [21].

## 2. Materials and Methods

### 2.1. Plasmids

The vector pEBTetLNC [22] was used to express the transporter cDNAs. pEBTetLNC is an Epstein–Barr replication plasmid for doxycycline-inducible protein expression in human cell lines based on the simple tetracycline repressor [23]. Expression was turned on by the addition of 1 µg/mL doxycycline to the culture medium. With ETT, for example, this system provides a high rate of transport of ET in the on-state (=100%) and a low rate (2–4%) in the off-state [23]. Insertion of the cDNA from pEBTetD/ETTh [23] yielded pEBTetLNC/ETTh. This cDNA codes for the wild-type carrier, with Leu at position 503.

The sequence of the open reading frame of the human CNT3 cDNA (gene symbol *SLC28A3*) corresponds to GenBank entry NM_022127. The 5′-interface between vector pEBTetLNC and cDNA is **AAACTT AAGCTT** gccacc ATGGAGCTGAGG (polylinker in bold, Kozak motif in lowercase, start codon underlined), and the 3′-interface is ACATTTTGATAG **GCGGCCGCGGGGCAG** (cDNA underlined, polylinker in bold). 

### 2.2. Cell Culture

In total, 293 cells (ATCC CRL-1573, also known as HEK-293 cells), a transformed cell line derived from human embryonic kidney, were grown in plastic culture flasks (Falcon 353112, Becton Dickinson, Heidelberg, Germany) at 37 °C in a humidified 5% CO_2_ atmosphere. The growth medium was Dulbecco’s modified Eagle medium (Life Technologies 31885-023, Invitrogen, Karlsruhe, Germany), supplemented with 10% fetal bovine serum (Biochrom, Berlin, Germany), 100 U/mL penicillin, and 0.1 mg/mL streptomycin (P4458, Sigma-Aldrich, Munich, Germany). The medium was changed every 2–3 days, and the culture was split every 5 days.

Stably transfected cell lines were generated as reported previously [23] using however TurboFect™ transfection reagent (R0532, Thermo Scientific, Dreieich, Germany). Since pEBTet-derived vectors [22,23] are propagated episomally, we used cell pools rather than single-cell clones. The cell culture medium always contained 3 µg/mL puromycin (P-600, Gold Biotechnology, St. Louis, MO, USA) to maintain plasmids. To turn on protein expression, cells were cultivated for at least 20 h with 1 µg/mL doxycycline (195044, MP Biomedicals, Eschwege, Germany) in the growth medium.

### 2.3. Transport Assays

Transport was assayed with stably transfected 293 cells. To measure initial rates of solute uptake, nearly confluent cell monolayers in paired dishes with and without transporter expression were incubated at 37 °C with unlabeled solute in a physiological uptake buffer. After washing and methanol lysis of cells, the solute content was determined by LC–MS/MS.

In more detail, cells were seeded in 60 mm diameter polystyrol dishes (83.3901, Sarstedt, Nümbrecht, Germany; precoated with 0.1 g/L poly-L-ornithine in 0.15 M boric acid-NaOH pH 8.4) and grown to a confluence of at least 70%. **Uptake buffer** contained 125 mM NaCl, 25 mM HEPES-NaOH pH 7.4, 5.6 mM (+)glucose, 4.8 mM KCl, 1.2 mM KH_2_PO_4_, 1.2 mM CaCl_2_, and 1.2 mM MgSO_4_. After preincubation of the cells at 37 °C for at least 20 min in 4 mL of uptake buffer, the buffer was replaced with 2 mL of the substrate in uptake buffer. Cells were incubated for the indicated times at 37 °C, washed four times each with 4 mL ice-cold uptake buffer, lysed with 1 mL of methanol, and then stored at −20 °C. The protein content of MS samples was estimated from 3 paired dishes; here, 0.1% *v*/*v* Triton X-100 in 5 mM TRIS-HCl pH 7.4 was used as lysis buffer. Protein was measured by the BCA (bicinchoninic acid) assay (Pierce; Thermo Scientific 23225, Life Technologies, Darmstadt, Germany), with bovine serum albumin as standard.

### 2.4. LC–MS/MS

After centrifugation of thawed cell lysates (2 min, 16,000× *g*, 20 °C), 20 µL (for the Atlantis HILIC column) or 10 µL were analyzed by HPLC, coupled to a triple quadrupole mass spectrometer (4000 Q TRAP, AB Sciex, Darmstadt, Germany). The following LC conditions were used (Shimadzu SLC-20AD Prominence HPLC): **cytarabine**, ZIC-HILIC column (5 μm, 2.1 × 100 mm; di2chrom, Merck, Darmstadt, Germany); A: 0.1% formic acid, B: 0.1% formic acid in acetonitrile; gradient flow, 0.2 mL/min: 85% B at 0 min, 85% B at 0.5 min, 30% B at 6 min, 30% B at 7 min, 85% B at 14 min, 85% B at 15 min; **gemcitabine**, like cytarabine, but with a different gradient, 0.4 mL/min: 85% B at 0 min, 85% B at 0.5 min, 30% B at 4 min, 30% B at 7 min, 85% B at 9 min, 85% B at 10 min; **gemcitabine triphosphate**, XBridge Shield RP18 column (5 µm, 3.0 × 100 mm; Waters, Dublin, Irland); A: 10 mM ammonium acetate pH 8.9, B: methanol; gradient flow, 0.2 mL/min: 15% B at 0 min, 15% B at 0.5 min, 85% B at 3 min, 85% B at 5 min, 15% B at 7 min, 15% B at 9 min; **ergothioneine,** Atlantis HILIC silica column (5 µm, 3.0 × 50 mm; Waters); A: 0.1% formic acid, B: 0.1% formic acid in acetonitrile; gradient flow, 0.4 mL/min: 90% B at 0 min, 90% B at 0.25 min, 10% B at 2 min, 10% B at 4 min, 90% B at 5 min, 90% B at 6 min; **TDC**, see gemcitabine; **uridine**, ZIC-pHILIC column (5 µm, 2.1 × 100 mm; SeQuant, Merck, Darmstadt, Germany); A: 10 mM ammonium acetate pH 8.9, B: methanol; gradient flow, 0.2 mL/min: 80% B at 0 min, 80% B at 1 min, 20% B at 4 min, 20% B at 5 min, 80% B at 9 min, 80% B at 10 min.

Atmospheric pressure ionization with positive (pos) or negative (neg) electrospray and ion detection was used. For quantification, the optimal collision energy for nitrogen-induced fragmentation in the second quadrupole was determined for each analyte. From the product ion spectra, the following fragmentations were chosen for selected reaction monitoring (m/z parent, m/z fragment, collision energy (V), ion mode): **cytarabine**: 244, 112, 17, pos; **ergothioneine**: 230, 127, 27, pos; **gemcitabine**: 264, 112, 25, pos; **gemcitabine triphosphate**: 502, 159, 30, neg; these m/z values agree with a previous report [24]; **TDC**: 244, 128, 13, pos; **uridine**: 243, 110, 24, neg. 

For each analyte, the area of the intensity vs. time peak was integrated. Linear calibration curves were constructed (weighting 1/y^2^) from at least six standards, which were prepared using control cell lysates as solvent. Sample analyte content was calculated from the analyte peak area and the slope of the calibration curve.

### 2.5. Calculations and Statistics

Results are presented, if not indicated otherwise, as the arithmetic mean ± SEM, with at least *n* = 3. All assays were at least performed two times, on separate days. An unpaired *t*-test was used to test for significance; two-tailed *p* values are given.

The graphs shown in time courses were estimated by non-linear regression using the function y = k_in_/k_out_ * c_out_ * [1 − exp(−k_out_ * x)], where c_out_ is the extracellular substrate concentration and k_in_, and k_out_ are rate constants.

As explained in detail previously [25], the transport efficiency (TE) was calculated as v/S (where v represents the initial rate of transporter-mediated uptake, and S is the substrate concentration). This TE is also known as clearance since it denotes the virtual volume of incubation buffer, which is cleared completely from the substrate by the transport activity per unit time [26].

### 2.6. Materials

Cytarabine (AB249843; abcr, Karlsruhe, Germany), L-(+)-ergothioneine (THD-201; Tetrahedron, Vincennes, France), gemcitabine (AB436344; abcr), TDC (169557-13-5; BOC Sciences, Shirley, NY, USA), uridine (0714.1; Carl Roth, Karlsruhe, Germany). All other chemicals were at least of analytical grade.

## 3. Results

### 3.1. Transport of Gemcitabine by CNT3

Our first aim was to express a prototype nucleoside transporter to see whether uptake of nucleosides in 293 cells can be measured by LC–MS/MS. Heterologous expression of human ENT1 in our system did not result in an increase in the uptake of uridine (not shown), compared with control cells (without expression). In contrast, the expression of the Na^+^-driven human concentrative nucleoside transporter 3 (CNT3) significantly increased the uptake of uridine (100 µM, 4 min, or 10 min) right away (not shown).

**Figure 1:**
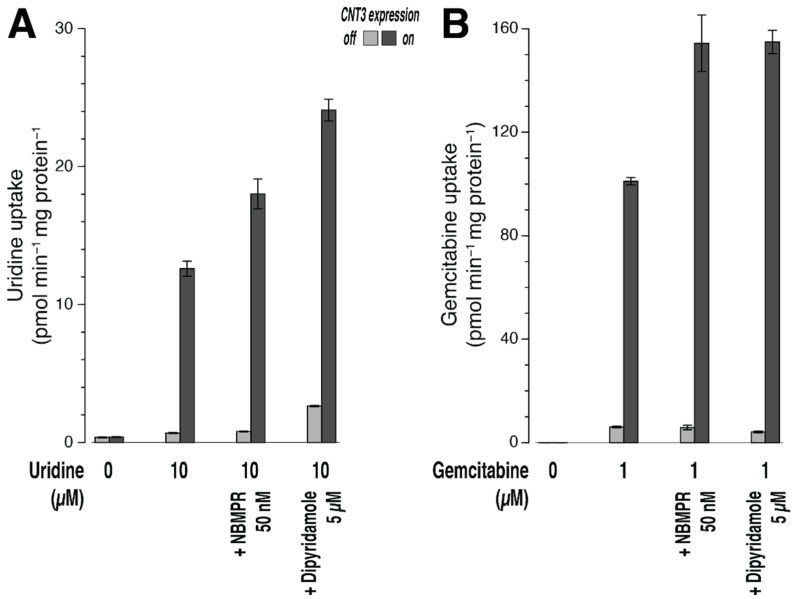
Uptake of uridine (**A**) or gemcitabine (**B**) into 293 cells with or without expression of CNT3. Stably transfected 293 cells with (on) or without (off) expression of CNT3 from humans were incubated at 37 °C with 10 µM uridine (10 min) or 1.0 µM gemcitabine (5 min) (± inhibitors as indicated) in uptake buffer or merely with uptake buffer, washed, and lysed with methanol. The nucleoside content of cell lysates (*n* = 3) was determined by LC–MS/MS.

It is known that 293 cells endogenously express the bidirectional carriers ENT1 and ENT2 (see the Section 1). These can adversely affect the signal-to-background ratio in uptake assays by adding to background uptake or by decreasing signal due to efflux of the substrate after uptake by CNT3. Since we had no clear preference, we tested the ENT inhibitors NBMPR and dipyridamole (see the Section 4 for inhibitor profiles). Uptake of uridine (10 µM, 10 min) was increased by expression of CNT3 by a factor of 19, from 0.68 ± 0.04 pmol min^−1^ mg protein^−1^ (control cells) to 12.6 ± 0.5 pmol min^−1^ mg protein^−1^ (Figure 1A). In paired background controls, after incubation without substrate, the signals were small, 0.40 ± 0.02 (CNT3 on) and 0.37 ± 0.04 pmol min^−1^ mg protein^−1^ (CNT3 off), respectively. In the presence of 50 nM NBMPR, the ratio of uptake rates, 18 ± 1 vs. 0.80 ± 0.02 pmol min^−1^ mg protein^−1^, was slightly better (f = 23). The difference corresponds to a transport efficiency *alias* clearance of 1.7 µL min^−1^ mg protein^−1^. In the presence of 5 µM dipyridamole, a factor of 9 was obtained, 24 ± 1 versus 2.6 ± 0.1 pmol min^−1^ mg protein^−1^. The difference corresponds to clearance of 2.1 µL min^−1^ mg protein^−1^. Thus, with uridine as a substrate, NBMPR would be the preferred ENT inhibitor.

With gemcitabine as substrate (1 µM, 5 min), a very high transporter-catalyzed accumulation of the unmodified compound was measured in the cell lysates (Figure 1B). Uptake in cells expressing CNT3 (101 ± 1 pmol min^−1^ mg protein^−1^) was 17-fold higher than that in the paired control cells (CNT3 off, 6.1 ± 0.3 pmol min^−1^ mg protein^−1^). In paired background controls, after incubation without substrate, the signals were negligible (Figure 1B), 0.019 ± 0.004 (CNT3 on) and 0.016 ± 0.001 pmol min^−1^ mg protein^−1^ (CNT3 off), respectively. In the presence of 50 nM NBMPR, the uptake of gemcitabine was even higher, 154 ± 11 vs. 5.9 ± 0.9 pmol min^−1^ mg protein^−1^, equivalent to a factor of 26. The difference corresponds to a clearance of 148 µL min^−1^ mg protein^−1^. In the presence of 5 µM dipyridamole, uptake was similar to that with NBMPR, 155 ± 5 vs. 4.1 ± 0.3 pmol min^−1^ mg protein^−1^. The difference corresponds to a clearance of 151 µL min^−1^ mg protein^−1^. Thus, despite the (expected) intracellular metabolism, uptake of non-metabolized gemcitabine into 293 cells by CNT3 could be detected easily by mass spectrometry even at a low gemcitabine concentration. NBMPR was chosen (see the Section 4) for the following experiments to improve the signal-to-background ratio.

### 3.2. Transport of Cytarabine by CNT3

**Figure 2:**
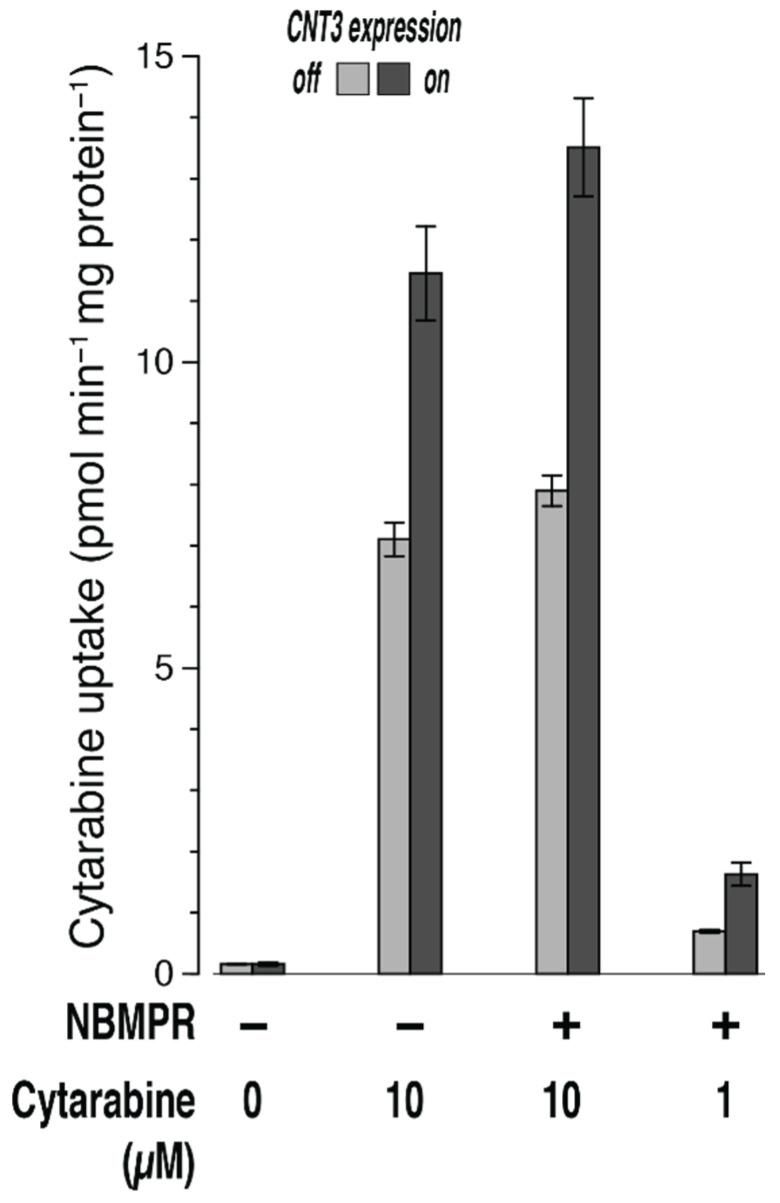
Uptake of cytarabine into 293 cells with or without expression of CNT3. Cells were incubated for 5 min at 37 °C with 1 or 10 µM cytarabine (plus 50 nM NBMPR as indicated) in uptake buffer or merely with uptake buffer. See legend in Figure 1 for further details.

According to the literature, cytarabine is not a substrate of CNT3 [27]. However, we observed even a small uptake by CNT3 here. The uptake of cytarabine (10 µM, 5 min) into cells expressing CNT3 was significantly (*n* = 3; *p* = 0.006) higher (11.5 ± 0.8 pmol min^−1^ mg protein^−1^) than that into control cells (expression off, 7.1 ± 0.3 pmol min^−1^ mg protein^−1^) (Figure 2). In the presence of 50 nM NBMPR, uptake was still slightly higher, at 13.5 ± 0.8 versus 7.9 ± 0.3 pmol min^−1^ mg protein^−1^ (*p* = 0.003). The latter difference corresponds to a clearance of 0.6 µL min^−1^ mg protein^−1^. Even with only 1 µM cytarabine (plus NBMPR), transport via CNT3 was still evident (Figure 2), at 1.6 ± 0.2 versus 0.70 ± 0.03 pmol min^−1^ mg protein^−1^ (*p* = 0.008). This difference corresponds to a clearance of 0.9 µL min^−1^ mg protein^−1^.

Cytarabine is just a stereoisomer of cytidine, and therefore, these are indistinguishable in LC–MS/MS. However, the measurement of cytarabine was not compromised by endogenous cytidine here, because in paired controls the content (presumably of cytidine) after incubation without substrate was markedly smaller than the values after incubation with cytarabine, at 0.16 ± 0.03 (CNT3 on) and 0.16 ± 0.01 pmol min^−1^ mg protein^−1^ (off), respectively (Figure 2).

We conclude that even the slow uptake of only 1 µM cytarabine by CNT3 into 293 cells could be detected by mass spectrometry.

### 3.3. Generation of Gemcitabine Triphosphate

Next, we wanted to measure the intracellular metabolism of gemcitabine in order to determine whether the expression of the ETT has an effect, as suggested by Sparreboom. Gemcitabine was chosen because (1) CNT3 affects rapid and high accumulation inside the cells and (2) as a fluorine compound, it has no endogenous stereoisomers that could interfere with MS quantitation of metabolites. Inside cells, gemcitabine is metabolized first by deoxycytidine kinase (dCK) to the monophosphate and then to gemcitabine triphosphate, the predominant metabolite [18]. Gemcitabine was used at 100 µM to generate sufficient triphosphate.

**Figure 3:**
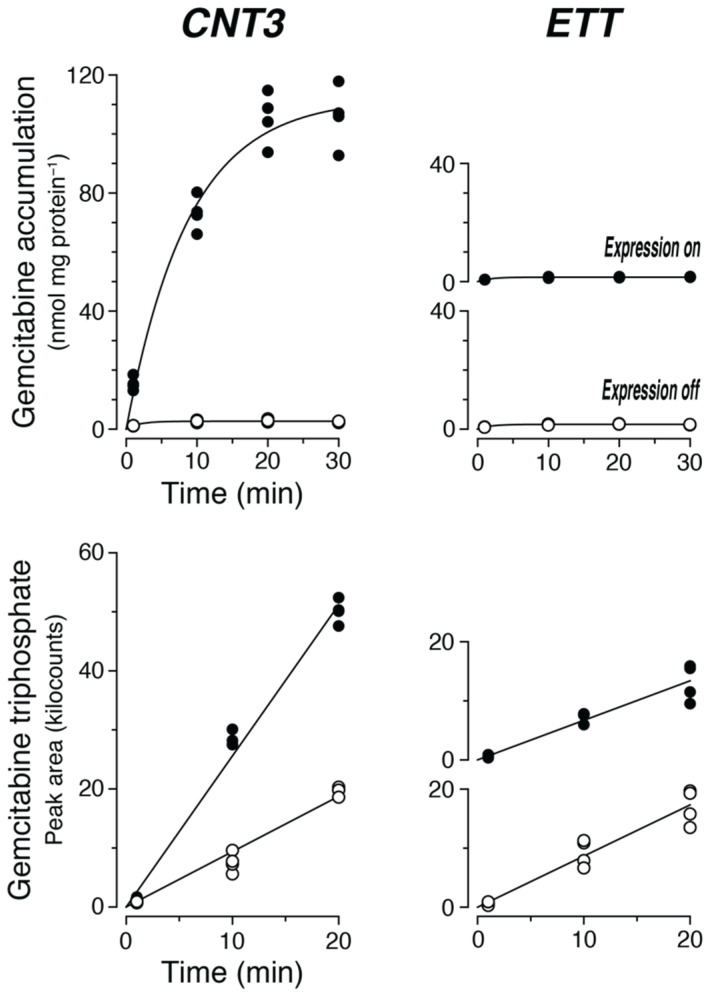
CNT3 catalyzes the uptake of gemcitabine into 293 cells and thus stimulates the generation of gemcitabine triphosphate; ETT does neither. Each circle represents a dish (*n* = 4). Time course of gemcitabine accumulation (**Upper row**). Cells without (open circles) or with transporter expression (filled circles) were incubated for the indicated times at 37 °C with 100 µM gemcitabine plus 100 nM NBMPR in uptake buffer. See legend in Figure 1 for further details. In the same lysates, gemcitabine triphosphate was measured by LC–MS/MS (**Lower row**). The peak area is proportional to analyte content. Calibration was not possible since we had no pure compound.

When cells expressing CNT3 were incubated with 100 µM gemcitabine (+ 100 nM NBMPR), a rapid increase in intracellular gemcitabine (k_in_ = 128 µL min^−1^ mg protein^−1^) was observed (Figure 3 top left). A plateau was reached after approximately 20 min. This level was circa 40-fold higher than in the paired control cells (CNT3 off). In parallel, the amount of gemcitabine triphosphate increased—to a good approximation—linearly (Figure 3, bottom left). In the cells with high gemcitabine (CNT3 on), the slope was 2.7-fold higher than that in the control cells.

Under the same conditions, expression of ETT—as expected—did not result in an increase in gemcitabine (Figure 3, top right) compared to control cells. Decisively, the synthesis of gemcitabine triphosphate (ETT on) occurred exactly (Figure 3, bottom right) as in the control cells (ETT off). The addition of a trace amount of ET (0.1 µM) verified the functional expression of ETT using the same lysates: Uptake into cells expressing the ETT was eightfold higher (without correction for endogenous ET; not shown) than uptake into control cells (ETT off).

We conclude that uptake of gemcitabine by CNT3 stimulates the generation of gemcitabine triphosphate in 293 cells. The expression of ETT, under the same conditions, does not increase the production of triphosphate.

### 3.4. Inhibition of Deoxycytidine Kinase

Finally, if the first step of gemcitabine metabolization catalyzed by dCK was a relevant pathway, then inhibition of this enzyme should increase intracellular gemcitabine levels. We used 2-thio-2′-deoxycytidine (TDC) as an inhibitor. To avoid acute inhibition of gemcitabine transporters by TDC, cells were pretreated for 1 h with 10 µM TDC in uptake buffer, washed twice with ice-cold buffer, and then incubated for 15 min with 10 or 100 µM gemcitabine (without TDC but with 100 nM NBMPR). To verify accumulation, TDC was measured in cell lysates made directly after washing; the intracellular concentration, calculated with a water space of 6.7 µL mg protein^−1^ [28], was 58 ± 1 (CNT3 off) and 992 ± 23 µM (CNT on). In paired controls after preincubation without TDC, the background was small, at 1.7 ± 0.4 and 1.3 ± 0.8 µM, respectively.

**Figure 4:**
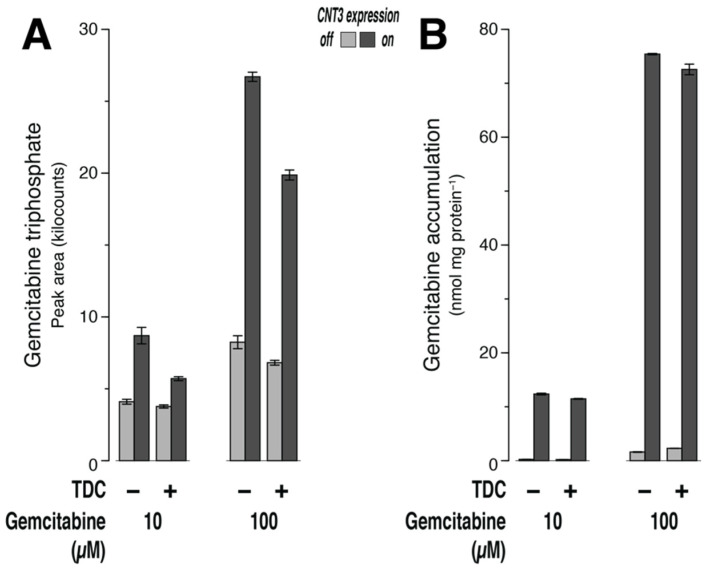
TDC inhibits the generation of gemcitabine triphosphate (**A**), but this does not increase gemcitabine (**B**). Cells without or with CNT3 expression were preincubated for 1 h with uptake buffer or with 10 µM 2-thio-2′-deoxycytidine (TDC) in uptake buffer, washed twice with ice-cold uptake buffer, and then incubated for 15 min at 37 °C with 10 or 100 µM gemcitabine + 100 nM NBMPR in uptake buffer. See legend in Figure 1 for further details.

Expression of CNT3 led to an increase in gemcitabine triphosphate production, as mentioned in Section 3.3 (Figure 4A). Of this increase, 58% (10 µM gemcitabine; *n* = 3; *p* = 0.01) and 29% (100 µM gemcitabine; *n* = 3; *p* = 0.0003) were inhibited by TDC, respectively. However, in the same dishes, inhibition of dCK did not lead to an increase in gemcitabine in the cells (Figure 4B).

We conclude that the inhibition of dCK did not cause an increase in gemcitabine. If the reaction catalyzed by dCK was a rapid outlet, then there should have been a surge.

## 4. Discussion

Based on radiotracer measurements, Sparreboom et al. have reported that the ETT transports the nucleosides cytarabine and gemcitabine with the highest efficiency (external Figure 5B) [8], as efficiently as ET. Verification in our heterologous expression system with LC–MS/MS quantification did not show the slightest transport activity [12]. Later, Sparreboom et al. confirmed our finding with their own MS analyses but again found high uptake with radioactive cytarabine (“Ara-C”) [16]. Sparreboom et al. explained this disparity as follows [16]: “… Ara-C can undergo rapid enzyme-mediated metabolism once inside cells to form mono-, di-, and tri-phosphorylated forms that may easily escape detection and result in underestimating the actual extent of uptake. The extensive formation of phosphorylated Ara-C metabolites was previously demonstrated in the HEK293 cells used in our experiments”. In other words, according to Sparreboom et al., intracellular metabolization of nucleosides is much faster than inward transport; therefore, the transported unmodified compounds cannot be detected in cell lysates by LC–MS/MS.

Our current experiments refute this hypothesis. To this end, we expressed the Na^+^/nucleoside cotransporter CNT3 (*SLC28A3*)—a prototype gemcitabine transporter—in the same cell line and verified its activity by uptake of uridine. In the course of our experiments, we sought to block the activity of endogenous ENT1 and ENT2 to a large extent in order to improve the signal-to-background ratio. Both transporters work bidirectionally. Inhibition could, therefore, improve the signal-to-background ratio in two ways: (1) creating less background by inhibiting the uptake of nucleosides via ENT1/ENT2 and (2) having more signal because, by inhibiting efflux via ENT1/ENT2 [21], less intracellular nucleoside is lost again after uptake via CNT3. NBMPR inhibits human ENT1 truly potently (K_i_ = approximately 1 nmol/L) but inhibits human ENT2 more than 1000-fold less so [20,29]. As a possible alternative, we tested dipyridamole, which inhibits ENT2 8 times more potently (K_i_ = 0.36 µmol/L) than NBMPR [20] and about 20 times more potently than dilazep [29] but ENT1 about 10 times weaklier than NBMPR [20,29]. The effects of both inhibitors (50 nM NBMPR or 5 µM dipyridamole) in measuring uptake of gemcitabine upon expression of CNT3 were similarly favorable here (Figure 1B). We subsequently used NBMPR because the 100-fold lower concentration, compared with dipyridamole made simultaneous inhibition of CNT3 or ETT less likely. Indeed, human CNT3 was not inhibited at all when expressed in *X. laevis* oocytes by 1 µM NBMPR [21] or by 10 µM NBMPR or 10 µM dipyridamole [30]. The putative uptake of cytarabine by the ETT was potently inhibited by dipyridamole (IC_50_ = 8 nM) but also by NBMPR (IC_50_ = 74 nM) (external Figure 3D in [8]). Accordingly, 5 µM dipyridamole must not be used for experiments with the ETT. However, in the presence of 50 nM NBMPR, more than half of the ETT activity would remain.

Using a radiotracer, high uptake of gemcitabine by CNT3 was previously observed in 293 cells at circa 233 µL min^−1^ mg protein^−1^ (corrected from 2% transfection efficiency to 100%) [21]. This translates to a clearance of 140 µL min^−1^ mg protein^−1^ in our system of uncorrected reporting if we assume 60% transfection efficiency. Our LC–MS/MS measurements (Figure 1B) in this study also revealed very high transport efficiencies of approximately 95 and 150 µL min^−1^ mg protein^−1^ for the substrate gemcitabine (without and with inhibition of the endogenous transporters ENT1/ENT2, respectively). If the hypothesis of ultrafast metabolization were true, then gemcitabine should not be detectable in the lysates. Thus, metabolization of the nucleosides in the 293 cells cannot be faster than very rapid inward transport.

Cytarabine was transported by ENT1 and ENT2 in a previous study but not by CNT1 and CNT3 [27]. Thus, at best, we expected very slow uptake by CNT3. By combining very low transport efficiency/clearance (<1 µL min^−1^ mg protein^−1^; cytarabine is clearly a poor substrate of the CNT3) and low concentration (1 µM), we generated a very slow inward flux of cytarabine. Nevertheless, we were still able to detect the transporter-catalyzed increase in the unaltered compound in the cells by LC–MS/MS (Figure 2). Thus, the metabolization of the nucleosides in the 293 cells cannot be faster than a very slow inward transport.

The lack of increase in gemcitabine triphosphate upon expression of the ETT (Figure 3) is further clear evidence against the hypothesis of (rapid) metabolization after transport. With CNT3 as a positive control for gemcitabine uptake, the increase was obvious. For a direct comparison, the metabolization of gemcitabine in the 293 cells to gemcitabine triphosphate was measured in parallel to the inward transport (Figure 3). Expression of CNT3 caused a rapid and high increase in intracellular gemcitabine until equilibrium after about 20 min. We have measured similar time courses in the past for nonmetabolized substrates such as ET [9], 1-methyl-4-phenylpyridinium [25], and TEA [31]. At this time point, approximately 40 times less gemcitabine was present in the control cells (CNT3 off). However, the rates of gemcitabine triphosphate production differed by only a factor of 2.7 (Figure 3). Thus, the intracellular enzymes could not process gemcitabine in proportion to its increase. To estimate the velocity of production of gemcitabine triphosphate (the pure substance was not available to us), we used—under identical HPLC conditions—the slope (320 ng^−1^) of the calibration curve for deoxycytidine triphosphate (dCTP). Gemcitabine triphosphate contains two fluorine atoms but is otherwise identical to dCTP. Accordingly, ca. 10 (CNT3 on) or 4 pmol min^−1^ mg protein^−1^ (CNT3 off) was produced. In parallel, the velocity of inward transport of gemcitabine was 12,800 pmol min^−1^ mg protein^−1^ (= k_in_ * 100 µmol/L). This (rough) estimate means that metabolization in these cells was much slower than inward transport, so there is time enough for accumulation of the unchanged substrate. In the study by Sparreboom et al. (external Figure 3E), cytarabine triphosphate was generated from 10 µM extracellular cytarabine with a velocity of ca. 4 pmol min^−1^ mg protein^−1^ [8]; again, the parallel uptake of cytarabine was much faster at ca. 1800 pmol min^−1^ mg protein^−1^.

Notably, we failed to monitor dCK (the enzyme that makes the first modification of gemcitabine and thus removes it from MS detection) activity here directly, because the concentration of nucleoside monophosphates is generally two orders lower than the triphosphates [18,32]. However, we could use the inhibitor TDC to block dCK. With a different cell line, this compound completely rescued cell growth at 10 µM (EC_50_ = 0.4 µM) by blocking the phosphorylation of cytotoxic cytarabine [33]. In order not to confound uptake of gemcitabine (15 min) by inhibition of CNT3, TDC (10 µM) was present in our experiments only during preincubation (1 h) and then removed from the outside by washing. In the uptake assay with 10 µM of gemcitabine, the production of gemcitabine triphosphate (above background, CNT3 off) was inhibited by 58% by TDC (Figure 4A). As would be expected for competitive inhibition, the effect was smaller (29% inhibition) with 100 µM gemcitabine but still evident. Importantly, this inhibition did not cause an increase in gemcitabine (Figure 4B). If the reaction catalyzed by dCK was a rapid outlet, then there should have been a surge.

The cause for the incorrect data of Sparreboom et al. remains unclear. Our previous speculation—namely, the inadvertent expression of a nucleoside transporter in addition to the ETT [12]—is now obsolete since Sparreboom et al. could not measure by LC–MS/MS any uptake of nucleosides by ETT either [16]. Perhaps the problem is in the purity or composition of the radiotracers.

To conclude, Sparreboom et al. have suggested that the intracellular metabolization of the nucleosides gemcitabine and cytarabine occurs so fast that the original compounds cannot be detected by LC–MS/MS after inward transport. Our current experiments clearly disprove this hypothesis. Even a very slow uptake of cytarabine into 293 cells was easily detected. The metabolization of gemcitabine was in fact much slower in these cells than inward transport by CNT3 such that the unchanged substrate accumulated to high levels. The fact, therefore, remains: the ETT does not transport nucleosides.

## Abbreviations

CNT3: concentrative nucleoside transporter 3; dCK, deoxycytidine kinase; ET, ergothioneine; ETT, ergothioneine transporter; LC, liquid chromatography; MS, mass spectrometry; NBMPR (*alias* NBTI; S-(4-Nitrobenzyl)-6-thioinosine); TDC, 2-thio-2′-deoxycytidine; TE, transport efficiency; TEA, tetraethylammonium.

## References

[1] Pastor-Anglada M., Perez-Torras S. (2018). Emerging Roles of Nucleoside Transporters. Front. Pharmacol..

[2] Boswell-Casteel R.C., Hays F.A. (2017). Equilibrative nucleoside transporters—A review. Nucleosides Nucleotides Nucleic Acids.

[3] Young J.D., Yao S.Y., Baldwin J.M., Cass C.E., Baldwin S.A. (2013). The human concentrative and equilibrative nucleoside transporter families, SLC28 and SLC29. Mol. Asp. Med..

[4] Young J.D. (2016). The SLC28 (CNT) and SLC29 (ENT) nucleoside transporter families: A 30-year collaborative odyssey. Biochem. Soc. Trans..

[5] King A.E., Ackley M.A., Cass C.E., Young J.D., Baldwin S.A. (2006). Nucleoside transporters: From scavengers to novel therapeutic targets. Trends. Pharmacol. Sci..

[6] Kose M., Schiedel A.C. (2009). Nucleoside/nucleobase transporters: Drug targets of the future?. Future Med. Chem..

[7] Vlachodimou A., Konstantinopoulou K., AP I.J., Heitman L.H. (2020). Affinity, binding kinetics and functional characterization of draflazine analogues for human equilibrative nucleoside transporter 1 (SLC29A1). Biochem. Pharmcol..

[8] Drenberg C.D., Gibson A.A., Pounds S.B., Shi L., Rhinehart D.P., Li L., Hu S., Du G., Nies A.T., Schwab M. (2017). OCTN1 Is a High-Affinity Carrier of Nucleoside Analogues. Cancer Res..

[9] Gründemann D., Harlfinger S., Golz S., Geerts A., Lazar A., Berkels R., Jung N., Rubbert A., Schömig E. (2005). Discovery of the ergothioneine transporter. Proc. Natl. Acad. Sci. USA.

[10] Gründemann D., Hartmann L., Flögel S. (2022). The ergothioneine transporter (ETT): Substrates and locations, an inventory. FEBS Lett..

[11] Gründemann D. (2012). The ergothioneine transporter controls and indicates ergothioneine activity—A review. Prev. Med..

[12] Tschirka J., Kreisor M., Betz J., Gründemann D. (2018). Substrate Selectivity Check of the Ergothioneine Transporter. Drug Metab. Dispos..

[13] Grigat S., Harlfinger S., Pal S., Striebinger R., Golz S., Geerts A., Lazar A., Schömig E., Gründemann D. (2007). Probing the substrate specificity of the ergothioneine transporter with methimazole, hercynine, and organic cations. Biochem. Pharmacol..

[14] Fork C., Bauer T., Golz S., Geerts A., Weiland J., Del Turco D., Schömig E., Gründemann D. (2011). OAT2 catalyses efflux of glutamate and uptake of orotic acid. Biochem. J..

[15] Schulz C., Fork C., Bauer T., Golz S., Geerts A., Schömig E., Gründemann D. (2014). SLC22A13 catalyses unidirectional efflux of aspartate and glutamate at the basolateral membrane of type A intercalated cells in the renal collecting duct. Biochem. J..

[16] Anderson J.T., Hu S., Fu Q., Baker S.D., Sparreboom A. (2019). Role of equilibrative nucleoside transporter 1 (ENT1) in the disposition of cytarabine in mice. Pharmacol. Res. Perspect.

[17] Buelow D.R., Anderson J.T., Pounds S.B., Shi L., Lamba J.K., Hu S., Gibson A.A., Goodwin E.A., Sparreboom A., Baker S.D. (2020). DNA Methylation-Based Epigenetic Repression of SLC22A4 Promotes Resistance to Cytarabine in Acute Myeloid Leukemia. Clin. Transl. Sci..

[18] Honeywell R.J., Giovannetti E., Peters G.J. (2011). Determination of the phosphorylated metabolites of gemcitabine and of difluorodeoxyuridine by LCMSMS. Nucleosides Nucleotides Nucleic Acids.

[19] Veltkamp S.A., Pluim D., van Eijndhoven M.A., Bolijn M.J., Ong F.H., Govindarajan R., Unadkat J.D., Beijnen J.H., Schellens J.H. (2008). New insights into the pharmacology and cytotoxicity of gemcitabine and 2′,2′-difluorodeoxyuridine. Mol. Cancer Ther..

[20] Ward J.L., Sherali A., Mo Z.P., Tse C.M. (2000). Kinetic and pharmacological properties of cloned human equilibrative nucleoside transporters, ENT1 and ENT2, stably expressed in nucleoside transporter-deficient PK15 cells. Ent2 exhibits a low affinity for guanosine and cytidine but a high affinity for inosine. J. Biol. Chem..

[21] Paproski R.J., Yao S.Y., Favis N., Evans D., Young J.D., Cass C.E., Zemp R.J. (2013). Human concentrative nucleoside transporter 3 transfection with ultrasound and microbubbles in nucleoside transport deficient HEK293 cells greatly increases gemcitabine uptake. PLoS ONE.

[22] Skwara P., Schömig E., Gründemann D. (2017). A novel mode of operation of SLC22A11: Membrane insertion of estrone sulfate versus translocation of uric acid and glutamate. Biochem. Pharmacol..

[23] Bach M., Grigat S., Pawlik B., Fork C., Utermöhlen O., Pal S., Banczyk D., Lazar A., Schömig E., Gründemann D. (2007). Fast set-up of doxycycline-inducible protein expression in human cell lines with a single plasmid based on Epstein-Barr virus replication and the simple tetracycline repressor. FEBS J..

[24] Veltkamp S.A., Hillebrand M.J., Rosing H., Jansen R.S., Wickremsinhe E.R., Perkins E.J., Schellens J.H., Beijnen J.H. (2006). Quantitative analysis of gemcitabine triphosphate in human peripheral blood mononuclear cells using weak anion-exchange liquid chromatography coupled with tandem mass spectrometry. J. Mass. Spectrom..

[25] Gründemann D., Liebich G., Kiefer N., Köster S., Schömig E. (1999). Selective substrates for non-neuronal monoamine transporters. Mol. Pharmacol..

[26] Schömig E., Lazar A., Gründemann D., Sitte H.H., Freissmuth M. (2006). Extraneuronal Monoamine Transporter and Organic Cation Transporters 1 and 2—A Review of Transport Efficiency. Handbook of Experimental Pharmacology—Neurotransmitter Transporters.

[27] Clarke M.L., Damaraju V.L., Zhang J., Mowles D., Tackaberry T., Lang T., Smith K.M., Young J.D., Tomkinson B., Cass C.E. (2006). The role of human nucleoside transporters in cellular uptake of 4′-thio-beta-D-arabinofuranosylcytosine and beta-D-arabinosylcytosine. Mol. Pharmacol..

[28] Martel F., Vetter T., Russ H., Gründemann D., Azevedo I., Koepsell H., Schömig E. (1996). Transport of small organic cations in the rat liver. The role of the organic cation transporter OCT1. Naunyn-Schmiedeberg’s Arch. Pharmacol..

[29] Visser F., Vickers M.F., Ng A.M., Baldwin S.A., Young J.D., Cass C.E. (2002). Mutation of residue 33 of human equilibrative nucleoside transporters 1 and 2 alters sensitivity to inhibition of transport by dilazep and dipyridamole. J. Biol. Chem..

[30] Ritzel M.W., Ng A.M., Yao S.Y., Graham K., Loewen S.K., Smith K.M., Ritzel R.G., Mowles D.A., Carpenter P., Chen X.Z. (2001). Molecular identification and characterization of novel human and mouse concentrative Na^+^-nucleoside cotransporter proteins (hCNT3 and mCNT3) broadly selective for purine and pyrimidine nucleosides (system cib). J. Biol. Chem..

[31] Gründemann D., Babin-Ebell J., Martel F., Örding N., Schmidt A., Schömig E. (1997). Primary structure and functional expression of the apical organic cation transporter from kidney epithelial LLC-PK1 cells. J. Biol. Chem..

[32] Zhang W., Tan S., Paintsil E., Dutschman G.E., Gullen E.A., Chu E., Cheng Y.C. (2011). Analysis of deoxyribonucleotide pools in human cancer cell lines using a liquid chromatography coupled with tandem mass spectrometry technique. Biochem. Pharmacol..

[33] Yu X.C., Miranda M., Liu Z., Patel S., Nguyen N., Carson K., Liu Q., Swaffield J.C. (2010). Novel potent inhibitors of deoxycytidine kinase identified and compared by multiple assays. J. Biomol. Screen..

